# *Bruguiera gymnorrhiza* (L.) Lam. Fruit Accelerates Healing in Gastric Injury via the Regulation of the NF-*κ*B Pathway

**DOI:** 10.1155/2022/1046712

**Published:** 2022-06-17

**Authors:** Xin Zhang, Jian-Hua Mai, Zhan-Wang Gao, Ling-Li Wang

**Affiliations:** ^1^School of Pharmaceutical Sciences, Guangzhou University of Chinese Medicine, No. 232, Waihuandong Road, Guangzhou Higher Education Mega Center, Guangzhou 510006, China; ^2^Graduate School, Guangzhou University of Chinese Medicine, No. 232, Waihuandong Road, Guangzhou Higher Education Mega Center, Guangzhou 510006, China

## Abstract

**Objective:**

The present study aimed at the anti-inflammatory and antioxidant effects of the extract of *Bruguiera gymnorrhiza (L.) Lam.* fruit (BGF) on the gastric injury.

**Materials and Methods:**

The chemical components in the extract of BGF were used in UPLC/Q-Orbitrap analysis. 60 SD rats were randomized into six groups: normal group (MC), ethanol-injured control group (EC), omeprazole group, and three groups with different doses (50, 100, and 200 mg/kg) of BGF. After continuous administration for seven days, the stomachs of rats were taken out to observe the pathological gastric tissue changes; inflammatory factors and oxidative stress markers in the stomach tissues were measured. Western blot (WB) analyses were conducted to explore the mechanism of BGF on gastric tissue and RAW 246.7 cells with excessive inflammation.

**Results:**

BGF enhanced gastric mucosal protection by improving the mucosal blood flow of the stomach and significantly decreased inflammatory factors and oxidative stress markers. Moreover, BGF significantly reduced the expression of p-NF-*κ*B p65. Consistently, BGF demonstrated similar effects on LPS-induced RAW 264.7 cells as it did in vivo.

**Conclusion:**

BGF could accelerate the healing of gastric injury by exerting antioxidant and anti-inflammatory effects and maintaining mucosal integrity.

## 1. Introduction

Ethanol-induced gastric injury is an acute or chronic gastric injury, resulting in the series of pathological changes, such as mucosal congestion, bleeding, and ulcers [1]. It is widely acknowledged that the pathogenesis of gastric injury involves an imbalance between aggressive and defensive factors [2, 3]. As an aggressive factor, alcohol causes substantial inflammatory reactions either by direct or indirect contact of ethanol metabolites with the mucosa, leading to impairment of the gastric mucosal defense [4]. It is known that the regulation of inflammatory cytokines expression and oxidative stress could improve the gastric mucosal defense [5].

Ethanol-induced gastric injury leads to substantial production of ROS, and excessive ROS can induce oxidative stress and mitochondrial depolarization, which triggers the damage to and apoptosis of gastric mucosal cells [6]. The lack of oxygen-free radical scavengers (SOD, CAT, and GPx) and high levels of lipid peroxides have been reported to aggravate the damage of cytotoxic injuries during gastric injury pathogenesis [7]. In this regard, ethanol could induce vasoconstriction in the gastric mucosa, reducing gastric mucosal blood flow (GMSF) [8]. A further reduction in blood flow to the gastric lining can cause damage to the gastric lining, leading to gastritis and ulcers. In the gastric mucosa, GMSF reduction is thought to be related to ethanol-induced prostaglandin E2 (PGE2) synthesis and inhibition of the NO synthesis as a result of decreased NOS activity [1]. At the same time, a high ROS level was involved in the inflammatory responses through NF-*κ*B cascade. In addition, previous studies have shown that the NF-*κ*B pathway regulates the synthesis of inflammatory cytokines, such as interleukin-6 (IL-6), tumor necrosis factor-*α* (TNF-*α*), and interleukin-1*β* (IL-1*β*), which will aggravate stomach damage and form a vicious circle [9, 10]. For these reasons, blocking the functional cross-talk between ROS and NF-*κ*B pathways might provide novel treatment approaches for gastric injury.

Currently, multiple drugs are available for the treatment of gastric injury [11]. Clinically, the long-term use of medications for treating gastric injury has significant side effects. Accordingly, the quest for alternative new therapeutic approaches has significant value. Nowadays, increasing attention has been focused on traditional Chinese medicine to treat gastric injury. TCM has been widely used in clinics to treat gastrointestinal-related diseases, with modern research demonstrating that Chinese medicine monomers, extracts, and compounds have gastroprotective effects [4, 11, 12]. These findings suggested that Chinese medicine plays substantial roles in treating gastric diseases.

In recent years, *Bruguiera gymnorrhiza* (L.) Lam. (BG) is an important tree species of mangroves, and it is a TCM material with a long history [13]. Besides, different parts of BG possess different pharmacological properties. It was recorded in some Chinese ancient books such as “Chinese Medicinal Flora,” “Compendium of Modern Materia Medica,” and other related books. For example, the decoction of the bark and root bark of BG can be used to clear away heat, detoxify, and relieve diarrhea for internal use and can be used to stop bleeding and reduce inflammation for external use; the leaves can be used to treat malaria; the fruits and hypocotyls can be used for diarrhea and sore throat [14]. People in other countries also have a lot of relevant medical experience with BG. For example, the BG leaves are used as anti-inflammatory and antibacterial and to treat burns in India; the BG fruits are used to treat shingles and eye diseases in Indonesia; the bark is utilized to treat malaria, and the roots and leaves are selected to treat burns [15]. Interestingly, the *Bruguiera gymnorrhiza* (L.) Lam. fruit (BGF) is used for folk medicine to mitigate chronic diarrhea and gastrointestinal inflammation [15, 16]. Previous studies have shown that BGF extract inhibits DSS-induced ulcerative colitis by activating the Keap1/Nrf2 signaling pathway [16]. Importantly, the cytokine level is regulated through NF-*κ*B transcription factors. The gastrointestinal system contains many organs, among which the stomach is intrinsically associated with the gut. Therefore, gastric injury may occur at the onset of gut disease [17]. To date, most studies on BGF have been mainly limited to the gut, and only few studies assessed its effect on the stomach. As a result, it remains unclear whether the beneficial function of BGF on gastric injury is because of inhibiting the NF-*κ*B pathway.

Therefore, the present study aims to evaluate the protective function of BGF on gastric injury, as well as elucidate the relevant mechanisms.

## 2. Materials and Methods

### 2.1. Reagents and Chemicals

Assay kits for SOD, MDA, CAT, GPx, and MPO were obtained from Jiancheng Bioengineering Institute (A001-3-2, A003-1-2, A007-1-1, H545-1-1, and A044-1-1) (Nanjing, China). ELISA assays for PGI, PGII, iNOS, NO, PGE2, TNF-*α*, IL-1*β*, and IL-6 were obtained from MEIMIAN (MM-70280R1, MM-70274R1, MM-0889R1, MM-70810R2, MM-0068R1, MM-0180R1, MM-0047R2, and MM-0190R1). Rabbit anti-NF-*κ*Bp65 conjugated antibody (AF5006), rabbit anti-p-NF-*κ*Bp65 conjugated antibody (AF2006), and mouse anti-*β*-actin antibody (T0022) were obtained from Affinity. Lipopolysaccharides (LPS) (batch number HY-D1056) were obtained from MedChemExpress.

### 2.2. Preparation of BGF Extracts

Fresh BGF (0.5 kg) was removed and crushed, and the powder was extracted three times with methanol for five days each. Methanolic extracts were concentrated by rotary evaporation (55°C, 0.08 MPA) and evaporated to dry in a water bath. The final weight of the dry extract was 39.1 g.

### 2.3. UPLC/Q-Orbitrap Analysis

The ultrahigh-performance liquid chromatography analysis was performed at 35C using UltiMate 3000 RS coupled with XB-C18 column (50 × 2.1 mm, 1.8 *μ*m). The elution gradient is outlined in Table 1. The flow rates were 0.3 mL/min, and the injection volume was 5 *μ*L. The high-resolution mass spectrometry analysis was performed on Q-Orbitrap with an electrospray ion source (ESI). The scan mode was set to positive and negative ion mode, full mass/dd-MS2 analysis, and the scan range was 150.0–2000.0 m/z. Data collected by the high-resolution UPLC/Q-Orbitrap system were retrieved and compared to databases (mzCloud, mzVault, and ChemSpider).

### 2.4. Cell Culture and Treatment

RAW 246.7 was purchased from Procell and was cultured in DMEM medium (Gibco, USA) and 10% fetal bovine serum at 37°C and 5% CO_2_. RAW 246.7 cells (3 × 10^3^ cells/well) were seeded in 96-well plates, and the cytotoxicity of BGF was assessed using the CCK-8 method (Biosharp, China). Confluent cells were treated with different concentrations of BGF (50∼1000 *μ*g/ml). After 24 hours of incubation, CCK8 reagent was added, and CCK8 detection was performed after 4 hours of incubation.

### 2.5. Animals and Grouping

6- to 8-week-old male SD rats were obtained from the medical experimental animal center of Guangdong Province (certificate of conformity: SCXK-2018-0002). The rats used in this experiment were approved by the animal ethics committee of Guangzhou University of Traditional Chinese Medicine (20201229002).

Rats were subdivided into six experimental groups of 12 animals each, namely, normal control group (NC), ethanol model control group (MC), omeprazole (OMEP) group (20 mg/kg), low-dose BGF group (BGFL, 50 mg/kg), medium-dose BGF group (BGFM, 100 mg/kg), and high dose BGF group (BGFH, 200 mg/kg). One hour after the last administration, except the normal group, the rats in others group were intragastrically administered with 5 ml/kg absolute ethanol [2]. Animals were anesthetized 1 hour after the last ethanol delivery.

### 2.6. Evaluation of Gastric Mucosal Lesions

The stomach tissues were cut open along the greater curvature and washed three times using a cold saline solution. The stomachs were fully expanded and photographed. The amount of gastric injury was observed by an independent viewer, and gastric mucosal lesion areas were measured using vernier calipers and expressed as the gastric ulcer index (UI).

The grading criteria for gastric mucosal injury are outlined in Table 2.

The calculating formula: UI = ∑*A*+∑(*B*×*C*).

### 2.7. Histopathologic Evaluation

Tissue samples were fixed by immersion in 4% paraformaldehyde. The gastric tissue sections were stained with HE. The pathological changes of gastric slices were observed by light microscope.

### 2.8. Determination of PGI, PGII, iNOS, NO, and PGE2 Levels

Tissues was minced, weighed, and homogenized in 0.9% saline (1 : 9, w/v) on ice to obtain a homogenate. The homogenate was centrifuged, and the tissue supernatant was collected for further assay.

The levels of PGI, PGII, NO, iNOS, and PGE2 in gastric tissue homogenate were analyzed using double-antibody sandwich method-specific ELISA kits.

### 2.9. Determination of Oxidative Stress

SOD, MDA, CAT, and GPx levels were detected using ELISA kits. Final results are expressed in U/mg, nmol/mg, and U/mg.

### 2.10. Determination of Inflammatory Cytokines

Serum TNF-*α*, IL-1*β*, and IL-6 levels were analyzed using ELISA kits according to the manufacturer's instructions. The absorbance (OD value) was measured and used a standard microplate reader and calculated from the standard curve.

### 2.11. Western Blot Analysis

Tissues was lysed with RIPA and was boiled for 10 min prior to centrifugation. Anti-p-p65 antibody and anti-p65 antibody at 1 : 1000 dilution were incubated overnight at 4°C and washed 5 times with TBST for 5 minutes each. Incubate with 1 : 500 diluted secondary antibody for 1 hour and monitor with ECL reagent. Finally, the quantification used ImageJ software.

### 2.12. Statistical Analysis

Statistical analysis was performed using SPSS 24 software. Between-group comparisons were made using one-way ANOVA (Dunnett T3). Data were presented as mean ± standard deviation ($\bar{x}$ ± S).

## 3. Results

### 3.1. UPLC/Q-Orbitrap Analysis of the Chemical Composition of BGF

**Figure 1:**
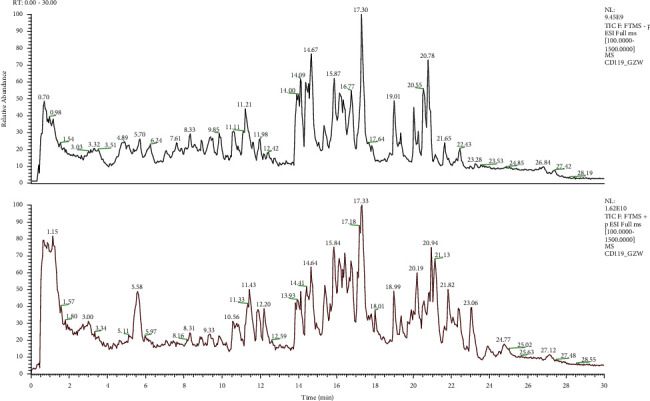
Detection of methanol extracts from BGF by UHPLC/Q-TOF-MS total ion chromatography (TIC).

The extract samples were matched to a total of 994 compounds from the mzCloud online database, of which 46 compounds had mzCloud best match scores greater than 90 (see Table 3). The positive and negative ion mass spectra of methanol extracts of BGF are shown in Figure 1.

### 3.2. BGF Inhibited Gastric Mucosal Lesions

**Figure 2:**
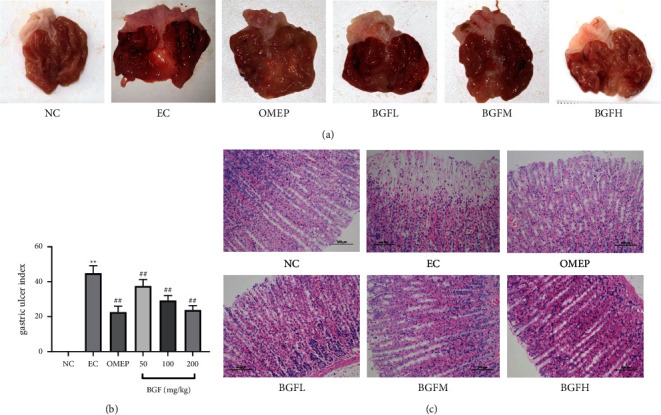
Evaluation of BGF inhibits gastric injury. (a) Gross images of the stomach. (b) The gastric ulcer injury index. (c) Histological photograph of gastric mucosa (100x magnification). ^*∗∗*^*P* < 0.01 vs. NC group; ^#^*P* < 0.05 and ^##^*P* < 0.01 vs. EC group.

To assess the treatment efficacy of ethanol-induced gastric injury with BGF, the stomachs were photographed and the gross pathological findings were observed. As shown in Figure 2(a), gastric lesions such as red punctate hemorrhages, thicker linear hemorrhage areas, and erosions appeared in the EC group compared with the NC group. However, the BGF pretreated and omeprazole groups presented with reducing lesions (redness and bleeding points) compared to the EC group. Similarly, BGFL pretreatment is almost like the omeprazole group records. The gastric mucosal injury was quantified by the gastric injury index. The EC group had a significantly higher gastric injury index than the NC group. A significantly reduced gastric injury index was observed in the BGF pretreated and omeprazole groups compared to the EC group.

### 3.3. BGF Significantly Attenuated the Pathological Gastric Damage

As shown in Figure 2(b), in the NC group, the gastric mucosa of rats was intact, with large amounts of tightly arranged glands and little connective tissue, chief cells, and parietal cells with cytoplasmic staining also being visible. The histopathology showed gastric mucosal epithelial cell necrosis and degeneration, with karyolysis and disappearance of the nucleus; cells in deeper layers were arranged irregularly while the submucosa appeared hyperemic, edematous, and was extensively infiltrated by inflammatory cells in the EC group. However, the obtained results were in a dose-dependent manner. The structure of gastric mucosal glands was relatively intact, with reduced loss of gastric mucosal epithelial cells and inflammatory cell infiltrates in the BGF pretreated group compared with the EC group. The histopathological slides demonstrated that the BGFH pretreated group experienced a similar therapeutic effect to the OMEP group.

### 3.4. Levels of PGI and PGII

**Figure 3:**
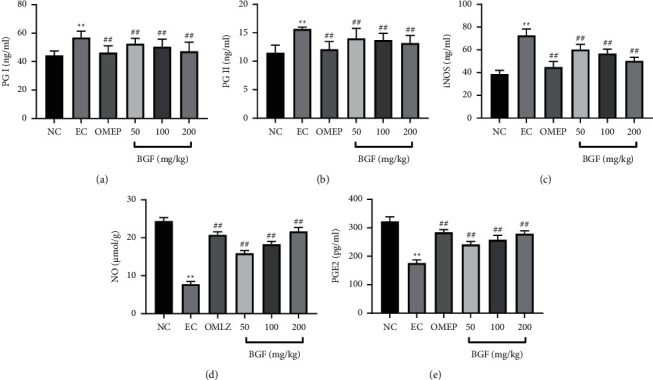
(a–e) The role of BGF in gastric injury-related protective factors in rats. ^*∗∗*^*P* < 0.01 vs. NC group; ^#^*P* < 0.05 and ^##^*P* < 0.01 vs. EC group.

Pepsinogen can be categorized as PGI and PGII, usually used for gastric disease screening and gastric mucosa monitoring. The expressions of PGI and PGII were significantly increased in the EC group compared with the NC group (Figure 3). Moreover, a significant reduction in levels of PGI and PGII was found in the BGF pretreated groups and omeprazole groups, compared to that of the EC group.

### 3.5. Levels of iNOS, NO, and PGE2

NO and PGE2 are potentially important gastric mucosal protection factors. As shown in Figure 3, significantly reduced NO and PGE2 levels and increased iNOS levels were found in the EC group. The BGF pretreated and omeprazole groups expressed significantly increased NO and PGE2 levels and decreased iNOS levels compared to the EC group.

### 3.6. BGF Inhibited Oxidative Stress Expression

**Figure 4:**
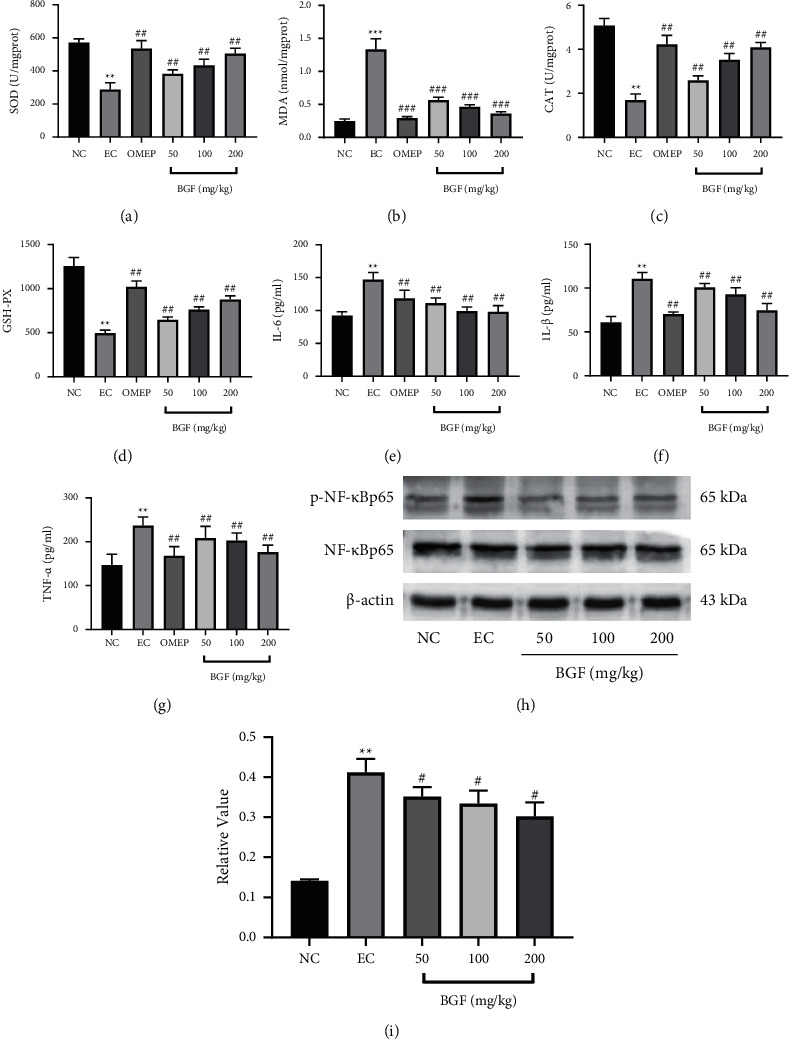
The BGF effects on levels of oxidative stress-related factors and inflammasome factors. (a–g) Effects of commutator on the NF-*κ*B signaling pathway in rats. (h) The levels of NF-*κ*B p65 and p-NF-*κ*Bp65. (i) The relative value of p-NF-*κ*B p65/NF-*κ*Bp65. ^*∗∗*^*P* < 0.01 vs. NC group; ^#^*P* < 0.05 and ^##^*P* < 0.01 vs. EC group.

As shown in Figures 4(a)–4(d), compared with the NC group, the EC group had significantly higher MDA levels and significantly lower SOD, CAT, and GPx activities (*P* < 0.05). Compared with the EC group, the MDA content in the pretreatment group and the omeprazole group was significantly decreased, and the activities of SOD, CAT, and GPx were decreased. These results suggested that BGF could reduce oxidative stress.

### 3.7. BGF Inhibited Inflammatory Cytokine Expression

As shown in Figures 4(e)–4(g), TNF-*α*, IL-1*β*, and IL-6 levels in the EC group were significantly increasing than those in the NC group. Compared with the EC group, the secretion of TNF-*α*, IL-1*β*, and IL-6 in the pretreatment group and the omeprazole group was significantly inhibited.

**Figure 5:**
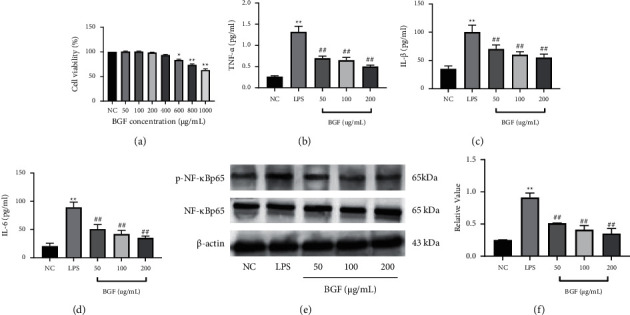
Inhibition of BGF on the NF-*κ*B pathway in vivo. (a) Effect of BGF on cell viability. (b) The BGF effects on levels of inflammasome factors: TNF-*α*, (c) IL-1*β*, and (d) IL-6. (e) The level of NF-*κ*B p65 and p-NF-*κ*Bp65. (f) The relative value of p-NF-*κ*B p65/NF-*κ*Bp65. ^*∗∗*^*P* < 0.01 vs. NC group; ^#^*P* < 0.05 and ^##^*P* < 0.01 vs. LPS group.

In CCK8 assay, as shown in Figure 5(a), BGF was not toxic in the concentration range of 50 to 400 *µ*g/ml. On this basis, RAW 264.7 cells were pretreated with 50, 100, and 200 *µ*g/ml BGF, and the expression levels of TNF-*α*, IL-6, and IL-1*β* were measured. In Figures 4(b)–4(d), the expressions of TNF-*α*, IL-1*β,* and IL-6 in the LPS group were significantly increasing compared to those in the NC group. Compared with the LPS group, the secretion of TNF-*α*, IL-1*β,* and IL-6 in the BGF group was significantly inhibited.

### 3.8. BGF Inhibited NF-*κ*B Signaling Pathway Activation

Given that the levels of TNF-*α*, IL-1*β,* and IL-6 are largely regulated by the NF-*κ*B signaling pathway, we evaluated the expression of p65 and p-p65 by western blot analysis to determine whether the gastroprotective effects of BGF occurred by inhibition of the NF-*κ*B signaling pathway. As shown in Figure 4(h), compared with the NC group, the expression level of p-p65 protein in the EC group was significantly increased, while the preconditioning rectification group and the omeprazole group were significantly decreased. When LPS treatment is compared with the NC group, p-p65 level was significantly increased in the LPS group, while the BGF treated group expressed significantly reduced p-p65 levels compared to the LPS group (Figure 5(e)). The results demonstrated that BGF could inhibit the inflammatory response by activating the NF-*κ*B pathway, thus exerting gastroprotective effects.

## 4. Discussion

It is widely acknowledged that gastric diseases affect the quality of life and have huge socioeconomic costs. Indeed, the pathogenesis of gastric injury involves an imbalance between aggressive and defensive factors [18]. Ethanol has been documented to dose-dependently or directly destroy the gastric mucosa barrier, resulting in decreased gastric mucus, sloughed mucosal epithelial cells, injured microvascular endothelial, and tissue ischemia or necrosis, which may lead to the formation of gastric mucosal ulcers [5]. Moreover, the ingestion of ethanol has been documented to increase the infiltration of inflammatory factors and oxidative stress in the stomach, leading to severe gastric injury [4, 7].

In the present study, the chemical composition and antioxidant and anti-inflammatory properties of BGF extract were determined. BGF has a variety of chemical compounds, among which stearic acid has an antiulcer effect. Previous studies have shown that palmitic acid and 12-oxo phytodienoic acid have significant anti-inflammatory effects, and afzelin has gastroprotective effects. Importantly, we found that gastric injury was significantly improved via BGF pretreatment for seven days. In short, BGF accelerated the healing of the gastric injury via its antioxidant and anti-inflammatory properties while maintaining mucosal integrity.

Based on previous studies, the gastric ulcer index has become an important indicator to assess treatment efficiency [19, 20]. Previous studies suggest that the ulcer index of the stomach was decreased significantly through BGF pretreatment for seven days, providing direct evidence of BGF inhibited gastric injury. Histopathological results showed that ethanol caused severe damage to the gastric mucosa, manifesting as necrosis and degeneration of epithelial cells, hyperemia and edema of the submucosa, and infiltration of inflammatory cells. This was consistent with the results of previous studies. Furthermore, our findings showed that BGF pretreatment could reverse ethanol-induced pathological changes; additional evidence showed that BGF inhibited ethanol-induced gastric injury. Pepsinogens (PG I and PG II) have been shown to be reliable biomarkers for gastric diseases [21]. Previous studies suggest that PG I and PG II levels increased significantly when erosions/ulcers developed in the stomach [22, 23]. Our results showed that PG I and PG II levels were significantly increased in the ethanol-induced rat gastric injury model, consistent with previous studies. However, expression of PG I and PG II was reduced after BGF pretreatment for seven days, suggesting that BGF plays a protective role in gastric injury.

It has been extensively documented that NO and PGE2 play important roles in the second mucosal defense system by maintaining gastric mucosal integrity [22]. NO is well known to exert gastroprotective effects and affect gastric mucosal cells by increasing vasodilation and enhancing mucin secretion [12]. NO can also regulate gastric acidity and secretion of PGE2. Interestingly, PGE2 can lead to increased secretion of mucus and NAHCO_3_, increased blood flow, and vasodilation, suggesting that PGE2 acts as a defensive factor and plays an important role in maintaining the integrity of gastric mucosa [10, 24]. This study demonstrated that NO and PGE2 levels were decreased in ethanol-induced gastric injury, and BGF pretreatment reversed this effect. Our results showed that BGF played a protective role in maintaining gastric mucosa integrity.

The severity of gastric injury correlates with the amount of superoxide anion. Superoxide anion can generate MDA through lipid peroxidation of cell membranes, and MDA has been shown to be an indicator of the end product of lipid peroxidation metabolism; excess MDA can produce a series of cytotoxic effects. Although SOD is an oxygen radical scavenger, it catalyzes the conversion of superoxide anion to hydrogen peroxide (H_2_O_2_) and O_2_, which is further oxidized [25]. Herein, the results showed that, in ethanol-induced gastric injury, BGF increased the expressions of SOD, GPx, and CAT, while decreasing MDA levels in a dose-dependent manner.

Many studies have shown that ethanol could induce a serious inflammatory response in gastric mucosal epithelial cells, which activate the NF-*κ*B pathway [23]. Activation of NF-*κ*B is known to trigger proinflammatory pathways leading to the excessive release of proinflammatory cytokines, which play significant roles in the inflammatory response. As a systemic inflammatory cytokine, TNF-*α* can activate neutrophils and lymphocytes to promote gastric damage and delay the healing of gastric lesions. Moreover, TNF-*α* can stimulate the infiltration of neutrophils into the gastric mucosa through iNOS and NF-*κ*B pathways [26]. Overproduction of IL-6 can lead to intravascular neutrophil activation and migration from the intravascular to the gastric epithelium; these could damage the gastric mucosa by inducing oxidative stress and releasing metabolites and enzymes [19].

Meanwhile, IL-6 has been documented to aggravate gastric mucosal injury by activating eosinophils, basophils, and monocytes. Mature IL-1*β* is a proinflammatory cytokine involved in the acute-phase response, can promote the recruitment of lymphocytes in gastric tissue, and amplify the acute inflammatory response when the immune cells are activated. Besides, activation of IL-1*β* is a major step of mediated proinflammatory responses. The present results suggested that the levels of IL-1*β* were increased, consistent with previous studies. Ethanol has been reported to increase the NF-*κ*B p65 level and downstream proinflammatory factors, suggesting that ethanol-induced gastric injury occurred through NF-*κ*B pathway [27]. Consistently, in the present study, BGF may exert a gastroprotective effect by inhibiting the NF-*κ*B signaling pathway and secretion of proinflammatory cytokines.

Excessive ROS can stimulate the inflammatory mediators NF-*κ*B, weaken its binding with I*κ*B, increase its content in the body, and produce large amounts of TNF-*α*, IL-1 *β*, IL-6, and iNOS, leading to a series of inflammatory reactions that can induce gastric tissue damage [28]. In the meantime, excessive ROS can also trigger activation of the NF-*κ*B pathway. Moreover, TNF-*α*, IL-1 *β*, and IL-6 can stimulate the production of mitochondrial ROS, thus creating a vicious cycle, in which excessive ethanol induces amplification of the inflammatory response [26]. The present results demonstrate that BGF has a positive regulatory effect on cytokines and exerts an antioxidative stress effect by inhibiting the amplification of inflammation in the blood circulation. Accordingly, BGF could potentially be used for the treatment of gastric injury disease to some extent.

In summary, we showed that BGF has therapeutic effects against gastric injury via activating the NF-*κ*B pathway.

## Figures and Tables

**Table 1:** The chromatographic gradient elution conditions.

Time (min)	The aqueous phase (%)	The organic phase (%)
0.0	98	2
1	98	2
5	80	20
10	50	50
15	20	80
20	5	95
25	5	95
26	98	2
30	98	2

**Table 2:** Grading criteria of gastric mucosal injury.

Gastric mucosal injury	1 point	2 points	3 points	4 points
Punctate mucosal erosions (*A*)	1	—	—	—
Length of injury (mm) (*B*)	1	3	5	＞5
Width of injury (mm) (*C*)	1-2	≥2	—	—

**Table 3:** The main fraction compound in the methanol extracts of BGF.

Compound name	Formula	Molecular weight	RT (min)	mzCloud best match
Stearic acid	C_18_ H_36_ O_2_	284.27135	21.637	99.5
Palmitic acid	C_16_ H_32_ O_2_	256.23973	20.532	98.8
12-Oxo phytodienoic acid	C_18_ H_28_ O_3_	274.19261	16.073	96.3
Afzelin	C_21_ H_20_ O_10_	432.10491	11.975	95.9
L-phenylalanine	C_9_ H_11_ NO_2_	165.07877	2.638	95.8
16-Hydroxyhexadecanoic acid	C_16_ H_32_ O_3_	272.23506	17.247	95.6
Adenosine	C_10_ H_13_ N_5_ O_4_	267.0963	2.696	95.4
Choline	C_5_ H_13_ NO	103.09996	0.606	95.1
(+/-)12(13)-DiHOME	C_18_ H_34_ O_4_	296.23433	17.32	94.8
Dimethyl sebacate	C_12_ H_22_ O_4_	230.15149	12.362	94.7
D-*α*-tocopherol	C_29_ H_50_ O_2_	430.38011	20.908	94.6
Quercetin	C_15_ H_10_ O_7_	302.04204	10.589	94.5
Epicatechin	C_15_ H_14_ O_6_	290.07883	7.041	94.4
Bis(2-ethylhexyl) phthalate	C_24_ H_38_ O_4_	390.2758	0.106	94.4
L-tyrosine	C_9_ H_11_ N O_3_	164.04724	1.348	94.3
Quercetin-3*β*-D-glucoside	C_21_ H_20_ O_12_	464.09477	10.585	94.3
N-phenyl-1-naphthylamine	C_16_ H_13_ N	219.10451	16.251	94.1
Myricetin	C_15_ H_10_ O_8_	318.03694	9.849	94
Rutin	C_27_ H_30_ O_16_	610.15211	10.586	93.9
Caprolactam	C_6_ H_11_ NO	113.0843	6.026	93.8
9-Oxo-10(E),12(E)-octadecadienoic acid	C_18_ H_30_ O_3_	294.21878	17.194	93.6
Indole-3-acrylic acid	C_11_ H_9_ N O_2_	187.0631	5.577	93.5
Isorhamnetin	C_16_ H_12_ O_7_	316.05783	11.489	93.1
9-Oxo-ODE	C_18_ H_30_ O_3_	294.21858	14.48	93.1
15-OxoEDE	C_20_ H_34_ O_3_	322.2504	18.255	93
Stearamide	C_18_ H_37_ NO	283.28685	20.575	93
Kaempferol	C_15_ H_10_ O_6_	286.04711	11.977	93
Hexadecanamide	C_16_ H_33_ NO	255.25564	19.411	93
DEET	C_12_ H_17_ NO	191.13091	12.705	92.9
4H-1-benzopyran-4-one, 6-*β*-D-glucopyranosyl-2,3-dihydro-5,7-dihydroxy-2-(4-hydroxyphenyl)-, (S)-	C_21_ H_22_ O_10_	434.12074	9.498	92.8
1-Linoleoyl glycerol	C_21_ H_38_ O_4_	336.26561	19.355	92.2
Bis(4-ethylbenzylidene)sorbitol	C_24_ H_30_ O_6_	414.20345	15.263	92.2
4-Phenylbutyric acid	C_10_ H_12_ O_2_	164.08344	20.906	91.9
*α*-Eleostearic acid	C_18_ H_30_ O_2_	278.22374	17.339	91.9
Ageratriol	C_15_ H_24_ O_3_	234.16158	13.961	91.7
(+/-)9-HpODE	C_18_ H_32_ O_4_	294.21925	15.502	91.5
9S,13R-12-oxophytodienoic acid	C_18_ H_28_ O_3_	292.20313	16.684	91.3
Corchorifatty acid F	C_18_ H_32_ O_5_	328.22444	13.969	91.3
Naringin	C_27_ H_32_ O_14_	580.17839	9.478	91.1
1-Dodecyl-2-pyrrolidinone	C_16_ H_31_ NO	253.24021	18.404	91
Cardamomin	C_16_ H_14_ O_4_	270.08893	13.016	90.9
Taxifolin	C_15_ H_12_ O_7_	304.05788	10.21	90.9
2-Aminooctadec-4-yne-1,3-diol	C_18_ H_35_NO_2_	297.26625	15.307	90.9
Nicotinic acid	C6 H_5_ N O_2_	123.03206	0.855	90.8
cis,cis-Muconic acid	C_6_ H_6_ O_4_	142.02657	5.095	90.7
3-Hydroxy-3,5,5-trimethyl-4-(3-oxo-1-buten-1-ylidene)cyclohexyl *β*-D-glucopyranoside	C_19_ H_30_ O_8_	386.19323	8.316	90.6
Dimethyl sebacate	C_12_ H_22_ O_4_	230.15149	13.721	90

## Data Availability

This study's data are included in the article, and the corresponding author can provide the primary data.
